# IPCRG is committed to lower cost, lower environmental impact and improved social impact: the triple bottom line in global primary care

**DOI:** 10.1038/s41533-021-00256-6

**Published:** 2021-11-08

**Authors:** Siân Williams, Ioanna Tsiligianni

**Affiliations:** 1 International Primary Care Respiratory Group, London, UK; 2 Department of Social Medicine, Faculty of Medicine, University of Crete, Heraklion, Greece

**Keywords:** Health care, Disease prevention

As the International Primary Care Respiratory Group (IPCRG) prepares for our 11th world conference, and with the 26th meeting of Conference of the Parties (COP26) approaching, the climate crisis is never far from our thoughts. Is meeting in person at a conference still culturally and ecologically acceptable? What are the likely unintended consequences of our decisions? What can we expect of each other with regard to our personal choices, professional behaviours and advocacy? Who are the experts we can learn from? Fortunately, the discussions are starting to lead to some clarity and improvement in our carbon literacy, but some questions still remain.

Firstly, as an organisation, in line with our “right care” approach, does IPCRG make it easy to act in an environmentally conscious manner? We were set up as a virtual organisation and have used online teleconferencing software since 2016, thus enabling flexible home-working and a low carbon footprint. However, during the COVID-19 pandemic we have also had to pivot educational programmes and most research online. We are now estimating the impact of this on our triple bottom line which takes into account the economic, environmental and social impact of our organisation^1^. Our significant reduction in travel over the last 2 years has had a positive impact on the environment and our finances, however, the social impact of this, and the impact on our network’s health and wellbeing is harder to quantify. Frustrations of bandwidth, difficulties in accommodating individuals across multiple time zones and a complete rewrite of educational content by very busy practising clinicians and academics has been stressful. Relationship building amongst early career researchers and members of our network has slowed down. There is a pressing need to develop and apply new relevant performance measures about the value of these networks^2^. On the positive side, phenomenal new low-cost technologies have emerged that improve the potential to connect globally and ensure no-one is left behind through translation, interpretation, transcription and recording. As we prepare to run our first in person conference for 4 years the challenge of ensuring hygiene standards during a pandemic and minimising the environmental impact of our meeting has come into sharp focus. It is unclear what responsibility we, as the organisers, should have in maximising the value of in person meetings vs virtual attendance, or in encouraging participants to “do the right thing” in terms of the carbon cost of their attendance. Practically-speaking, what does this mean for the provision of refreshments and how “waterwise” (https://www.waterwise.org.uk) we are?

Secondly, clinicians across the world are starting to test and evaluate the impact of integrating a green agenda into their consultations, and the respiratory community are highly engaged. For example, they are finding ways to encourage patients to walk safely rather than drive, and to ensure that patients want the inhalers that they are prescribed—and are capable of using them—to reduce emissions and plastic waste^3^. At the same time, they need to respond to concerns about trends in outdoor air pollution, changing pollen seasonality, poor housing, and occupational health to help the individual make decisions on the balance of risk. New decision-aids will be needed to enable estimation and communication of risk that also takes account of new technology, but in the meantime there are at least two guiding principles:

1. Primary care can deliver on the triple bottom line of lower cost, lower environmental impact and improved social impact compared to hospital care because it is embedded in local neighbourhoods and includes primary prevention as a core service. Therefore, it can significantly reduce the development of both communicable and non-communicable conditions, reduce patient and family travel cost and impact and, when it takes a public health approach, can integrate local intelligence about community assets such as social services, parks, cycle routes, community organisations and support groups into care planning^4^.
2. Primary care remains a huge contributor to the carbon footprint through pharmaceutical emissions (via manufacture and use), and patient and staff travel^3^. Prioritising prevention of long term conditions and tackling overdiagnosis and waste by taking a right care approach will reduce emissions and conserve scarce human and financial resources^3,5,6^.

Thirdly, the COVID-19 pandemic has not only reminded us that public health and individual health are entangled, but also that scientists and clinicians have an important advocacy role—to shape how politicians, peers and patients think about problems and solutions using the scientific evidence^7^. This role now needs to extend to the climate crisis and respiratory health. This advocacy might be about what research is funded and completed as well as what research is accelerated into practice or policy.

We already have good examples of research influence using primary care data. Our FRESH AIR protocol in four low- and middle-income countries combined strategies against tobacco and indoor biomass smoke, because communities are exposed to both and need holistic community- and person-centred research and solutions^8,9^. In Pakistan, our colleagues have previously presented their empirical knowledge about the impact of seasonal pollen on asthma in Islamabad. As part of RESPIRE, funded by the UK National Institute of Health Research (NIHR), they have commenced research to investigate the link between pollen, fungal spores and increased respiratory mortality^10^. The Malaysian team has performed an ecological analysis of asthma health outcomes and piloted an approach to determine the influence of potential environmental factors on asthma events^11^.

In terms of translating research into practice, colleagues in Bermuda have 10 years of experience running Pillows for Prevention to educate children and families about the relationship between the environment and asthma^12^. Open Airways delivers an annual pillow replacement programme for primary school children at high risk of asthma exacerbations due to exposure to dust mite and droppings and mould spores that thrive in high humidity.

In Australia, our colleagues have witnessed the impact on lung health of thunderstorms and bushfires which has revealed that many affected were not using inhaled corticosteroids^13–15^. The ready availability of over the counter short-acting beta agonists in Australia^16^ may lead to self-treatment of episodic symptoms. This creates over-reliance which leads to a vicious circle of high demand, and therefore positive reinforcement of an emergency behaviour. We need collective advocacy for appropriate policies and reimbursement to enable patients to receive, and pharmacists and GPs to deliver, right care because climate change means these events are likely to recur.

At the policy level, there are now many trusted sources that are collaborating to provide high quality communications material. These include the Global Climate and Health Alliance, which is an alliance of health non-governmental organizations, health professional organizations, and health and environment alliances from around the world, whom we have invited to speak at our 11th World Conference and whose letter we have signed: https://healthyclimateletter.net/, the World Health Organization (WHO), the United Nations Environment Programme (UNEP), the World Bank and the Climate and Clean Air Coalition, who created Breathelife (https://breathelife2030.org/resources/#health-professionals) to mobilize cities and individuals to protect our health and our planet from the effects of air pollution and provides a source of trusted communications material. Together with new WHO Air Quality Guidelines (AQG), based on convincing scientific evidence about the harms caused by exposure to low levels of conventional air pollutants, we have the information we need to advocate for change^17^. Our partners, the International Pharmaceutical Federation, have developed a call to action mobilising community pharmacists to mitigate air pollution^18^.

Let us also remember that this is WHO’s World No Tobacco Day—extended into a year-long campaign—focussed on helping people quit. This is probably one of the most powerful interventions that primary care can make to deliver the triple bottom line. In terms of social impact it will save lives, extend lives and improve the quality of life^19^. It will reduce street pollution through fewer discarded butts and lower tobacco production will lead to reduced deforestation, water use and carbon dioxide emissions^20^. The same primary care behaviour of making every contact count can also be applied to other preventive measures to reduce the growth in long term conditions and long term use of pharmaceuticals^21^.

In conclusion, we are trying to improve our carbon literacy and understand how to deliver the triple bottom line. While we have more learning to do, there are some actions we can take:

- For policy makers, invest in primary care and support the delivery of right care.
- At individual clinical level, help a person quit tobacco because it has enormous personal and planetary consequences.
- At individual clinical level, apply the same “making every contact count” brief behavioural intervention to physical activity, alcohol use and mental wellbeing.
- Record findings and actions in primary care electronic records routinely because aggregated primary care data on changes in seasonality, frequency and severity of symptoms and diagnosis and treatment outcomes can also be a powerful force for change.
- At practice level, commit to Asthma Right Care to reduce over-reliance on short-acting beta agonists and enable people with asthma to select the device that they will use and not waste.
- At community level, support everyone, including people with lifelong breathlessness to find ways to walk outside safely, to improve their cardio-pulmonary function and reduce emissions.
